# Design and methods of the Adult Inuit Health Survey 2007–2008

**DOI:** 10.3402/ijch.v71i0.19752

**Published:** 2012-11-16

**Authors:** Helga Saudny, Donna Leggee, Grace Egeland

**Affiliations:** 1 McGill University, School of Dietetics and Human Nutrition/Centre for Indigenous Peoples’ Nutrition and Environment (CINE), Ste Anne de Bellevue, Canada; 2 University of Bergen, Department of Public Health and Primary Health Care, Kalfarveien 31, 5018 Bergen, Norway

**Keywords:** International Polar Year, Inuit, health research, survey, Canadian Arctic

## Abstract

**Background:**

The Canadian International Polar Year (IPY) program made it possible to undertake much needed health research in 3 jurisdictions within the Canadian Inuit Nunangat (homeland) over a 2-year period: Inuvialuit Settlement Region (ISR), Nunavut Territory, and Nunatsiavut.

**Design:**

The Adult Inuit Health Survey (IHS) was a cross-sectional survey and provides baseline data upon which future comparisons can be made for prospectively assessing factors leading to the progression of chronic diseases among Canadian Inuit. With the help of the Canadian Coast Guard Ship *Amundsen*, which was equipped with research and laboratory facilities, 33 coastal communities were visited; land survey teams visited 3 inland communities.

**Results:**

The Adult IHS succeeded in obtaining important baseline information concerning the health status and living conditions of 2,595 adults living in ISR, Nunavut and Nunatsiavut.

**Conclusion:**

Information from this survey will be useful for future comparisons and the opportunity to link with the International Inuit Cohort, a follow-up evaluation, and for the development of future health policies and public health interventions.

Inuit are resilient, thriving, and adaptive people notwithstanding the rapid changes occurring across all dimensions of life, and in the face of the disparities in health and longevity that exist compared with other Canadians (1). The majority of Canadian Inuit reside in 4 regions across Inuit Nunangat or Inuit homeland, the Inuvialuit Settlement Region (ISR) (Northwest Territories), Territory of Nunavut, Nunavik (Northern Québec), and Nunatsiavut (Northern coastal Labrador), all representing land claim areas. Most live in coastal communities, and according to the 2006 Canadian Census, there were a total of 50,485 Inuit in Canada (2). The many health challenges faced by Inuit (3–10) require Inuit-specific approaches. To find solutions for these challenges as well as to inform public policy makers, the Inuit Health Survey (IHS) was designed and completed. A summary of the design and methods used in the IHS are presented in this article.

## Participatory research process

### Consultations and agreements

McGill University/Centre for Indigenous Peoples’ Nutrition and Environment (CINE) follows a framework of participatory research (11,12). Starting in 2006 and continuing until 2012, extensive consultations took place with stakeholders, community representatives, regional health officials, territorial government representatives, university partners, and the IPY-IHS team. Once steering committees were formed in each of the 3 jurisdictions, memoranda of agreements were developed outlining the roles and responsibilities prior, during, and after data collection. While each committee identified specific regional roles, some of the common topics included the governance of and access to data, short- and long-term storage of data, and obligations for follow-up to individual participants and communities. In addition, community–university agreements were developed, which invited the survey into the communities and identified the roles and responsibilities of the partners.

### Ethics and consent

Certification of Ethical Acceptability for Research Involving Human Subjects for the IHS was obtained from the McGill Faculty of Medicine Institutional Review Board in April 2007 with annual renewals until March 2013.

Scientific Research Licenses were obtained from the Nunavut Research Institute (Iqaluit, Nunavut) and from the Aurora Research Institute–Aurora College (Inuvik, Northwest Territories). The Nunatsiavut review board waived the requirement for a license because the IHS team had engaged in extensive participatory processes.

In recognition of Inuit's strong oral traditions, a “visual” consent form was created as a DVD in the appropriate Inuit languages (Inuktitut, Inuinnaqtun, Siglitin, Uummarmiutun, Nattilik, and Inutitut). The DVD followed the written consent form word-for-word and depicted all clinical and laboratory procedures. After watching the DVD, participants who consented to participate in the study signed the written consent form, on which they could also indicate whether or not they wanted to take part in the longitudinal follow-up as part of the larger International Inuit Cohort.

## Sampling strategy

### Sample size calculations

**Fig. 1:**
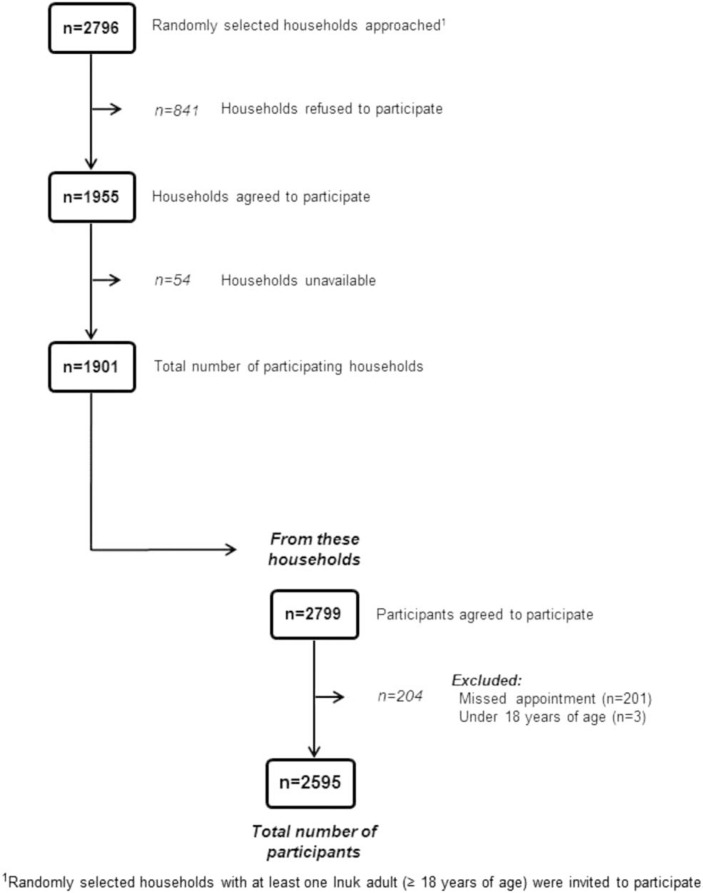
Flow chart of participating households and participants.

The survey was cross-sectional and used a random sampling of households in each community. Most communities provided the survey team with a list of houses, which were assigned sequential numbers. These lists were then used for randomly selecting households, using either a random digit table or a computerized random number generator. Households were contacted in the order that they appeared on the randomized list that was given to the head nurse of the land team. Within each household, all Inuit adults aged 18 years or older were eligible to participate. Pregnant women were excluded. The 2006 population estimates for the ISR, Nunavut and Nunatsiavut (13,14) were increased by 2% per year to account for population growth. The total estimated population of adults in the 3 survey regions in 2007 was 17,726 individuals. It was estimated that a sample of 12% of adults (or 40 individuals if 12% represented fewer than 40) in each community would produce an overall sample size of approximately 2,381, which was consistent with our goal of 2,000 participants, providing statistical power to identify the prevalence and correlates of health indicators. A total of 2,796 Inuit households were approached, 1,901 households (68%) participated with a total of 2,595 adults participating (Appendix A, Fig. 1).

### Assignment of participant and study numbers

Once a household member gave written consent to participate, he or she was given a 9-digit participant number. This number identified each person within the unique household and linked each person to the study year, the regional jurisdiction, and the community. The participant number plus first and last name, address, and gender were entered on a confidential community household list. When the participants arrived aboard the ship for their appointment, a file was opened for them and a sequential, anonymous study number was assigned to each participant. To protect confidentiality, any form which contained personal identifiers was removed from participants’ files and stored separately and securely; thereby only the unique study number was used for questionnaire items and laboratory results.

### Participant compensation

Each participant was given a $15 gift card to the community's grocery store regardless of whether they completed the entire survey or not. Participants were also entered into regional prize draws for gift certificates of $500 for 1st, and $100 for 2nd and 3rd prizes.

## Logistics

### Time frame

The survey took place over a 2-year period in late summer and fall of 2007 and 2008. Thirty-three coastal communities and 3 inland communities were visited. The survey began in Nunavut in August 2007 and continued until the end of September, visiting 18 communities. In 2008, the survey visited 6 communities in ISR, 7 communities in Nunavut, and 5 communities in Nunatsiavut between August and October.

### Preparation of communities

**Fig. 2:**
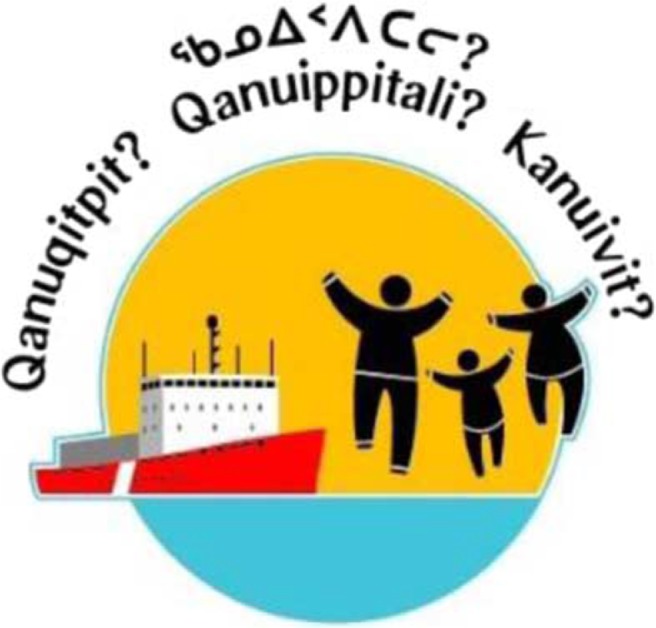
Inuit Health Survey (IHS) logo.

A promotional campaign, planned in consultation with our steering committees, was conducted to inform Inuit residents about the objectives and benefits of the survey and to encourage their active participation. Leaflets, posters, brochures, and a survey logo (Fig. 2) were designed in different Inuit dialects. Press releases were sent to regional newspapers before and during data collection. Local radio and cable TV advertisements featured the survey and promoted community awareness. Posters showing the ship's arrival in the community were displayed in grocery stores, community centers, health centers, and schools.

Community regional training events were held in Rankin Inlet, Iqaluit, and Inuvik for representatives from communities and land-team nurses and their assistants. Sessions covered the content of the survey, how to recruit participants and respond to commonly asked questions, instructions for meetings with Elders, mayors, and health centers, how to complete consent forms, household composition charts and 1 questionnaire and schedule clinic appointments.

Following these training events, 3 land teams, each consisting of a nurse and 2 assistants, traveled to the communities well ahead of the ship's schedule to inform the community, hire and train additional assistants, recruit participants, and conduct face-to-face interviews prior to the ship's arrival. Employers were contacted about the survey to encourage them to authorize time-off without loss of pay for survey participants. Three attempts were made to visit the home. If no one was home, pamphlets were left at the home with the research team's contact information. Refusals, no shows, and reasons for refusal were recorded.

A major challenge during the voyage was the logistics of moving 3 separate land teams to all participating communities well in advance of the ship's arrival and having participants ready for their appointments on the ship. Changing weather conditions posed another challenge as flights were cancelled or delayed in and out of communities thereby requiring flexibility and back-up plans. For example, nurses and community research assistants from the ship were at times moved to shore to facilitate fieldwork. Working very long hours to accommodate special situations in the communities (e.g. funerals or weddings) presented another challenge.

### Training of staff

The IHS provided extensive training opportunities for approximately 100 short-term staff each year representing community assistants, drivers, nurses, bilingual Inuit interviewers, participant liaison, laboratory technicians, quality control officers, research assistants, dieticians, specialists, and graduate students.

Interviewers working on the ship received extensive training to conduct detailed dietary assessments and face-to-face interviews on various dimensions of health. All interviewers received on-going training, as needed. Interviewers were instructed to read the questions as worded in the questionnaire, to offer clarification when requested, and not to ask leading questions. Bilingual interviewers also acted as greeters and interpreters, welcoming participants and ensuring their comfort and safety while on board the ship. They also translated information from nurses and specialists, which was especially important for Elders.

Licensed nurses responsible for clinic measurements (height, sitting height, weight, % body fat, waist circumference, blood pressure, and pulse) and blood samples were trained for the various procedures by an experienced clinic manager. A team of laboratory technicians was trained by an experienced laboratory manager to prepare blood samples for future analyses. All survey staff signed confidentiality agreements.

## Data collection

### Questionnaires

Survey participants completed a number of questionnaires. For each participating household, a principal respondent completed the household composition questionnaire and the home-based questionnaire for that household. The household composition enabled an assessment of household crowding, the number of persons living in the house, their ages, gender and relationship to the principal respondent. The home-based questionnaire, which was patterned after the 2004 Nunavik IHS (15) and the Canadian Community Health Survey cycle 2.2 (16), covered the home environment, living conditions, smoking behavior in the home, employment, income and expenses, food security and access to country food. Food security questions were derived from the USDA 18-item household food security survey module (17,18) and modified by Indian and Northern Affairs Canada (INAC) to improve acceptability among Inuit. All participants were asked to bring their medications to the clinic appointment to enable the nurses to complete the medicine and supplement use questionnaire.

Each participant completed an individual questionnaire, a 24-hour food recall, a food frequency questionnaire and a community and personal wellness questionnaire. To facilitate comparison with the Nunavik IHS, the questionnaires were developed using many of the same items and wording as that of the Nunavik survey (15). The questionnaires focused on general and dental health, family medical history, smoking habits, socio-demographic information, reproductive history for women, and physical activity (15,19,20). To assess physical activity, the short form version of the International Physical Activity Questionnaire was utilized (21). Community consultations indicated a strong preference for the use of the short form, which covered the last 7 days of physical activity to minimize research burden to participants. The community and personal wellness questionnaire was either self- or interviewer-administered and consisted of questions about mental health, alcohol and drug use, gambling habits, suicide, and violence and sexual abuse (13,22,23).

Diet was assessed through 24-hour dietary recalls, a comprehensive past-year traditional food frequency questionnaire, and an abbreviated market food frequency questionnaire. Due to survey logistical constraints, repeat 24-hour dietary recalls were not collected and a lengthy market food frequency questionnaire was not administered. The 24-hour food recall was administered using a multiple pass technique developed by the USDA (24), and while 1 recall is inadequate to characterize usual diet for individuals it can provide an estimate of dietary intake for a large study population. The food frequency questionnaire dealt with the consumption of common country food available in the 3 jurisdictions, including consumption when food items were in and out of season where seasonal availability was determined through community harvest calendars.

### Quality control

Two quality control officers checked all returned forms and questionnaires for completeness and oversaw ongoing training for interviewers, as required. Clinical equipment was checked daily according to the manufacturer's instructions.

## Clinic data

### Anthropometry

Height was measured to the nearest 0.1 cm with a portable stadiometer (Road Rod 214 Portable Stadiometer, Seca, MD). Sitting height was measured to the nearest 0.1 cm with a custom made sitting height table. Weight (to the nearest 0.1 kg), percent body fat and basal metabolic rate were measured on a bioelectrical impedance analysis instrument (Tanita TBF-300GS, Tanita Corporation of America Inc., Arlington Heights, IL). Shoes and socks were removed for all measurements and 500 g was subtracted for clothing weight. Waist circumference was measured, after expiration, at the midpoint between the top of the hip and the last loose rib to the nearest 0.1 cm with a cloth retractable tape measure. For participants with a pacemaker, weight was measured on a Seca scale (Medical Scale Model 214, Seca Corp., Ontario).

### Blood pressure and pulse

Blood pressure was measured to the nearest 1 mm Hg and pulse to the nearest beat per minute with a BpTRUTM Vital Signs Monitor (VSM MedTech LTD., Coquitlam, BC, Canada). Three readings, spaced 2 minutes apart, were recorded.

### Blood samples

Before a blood sample was taken, glycemia was determined with the OneTouch^®^ Ultra2TM glucometer (Life-Scan, Inc., Milpitas, CA). If the reading was greater than 7 mmol/L, the participant was not eligible for the oral glucose tolerance test (OGTT). All participants were asked to fast (water was allowed) for at least 8 hours, beginning at midnight prior to their appointment on the ship. Participants with morning appointments had fasting blood samples drawn on the ship, while participants with afternoon appointments on the ship had fasting blood samples drawn in their homes in the morning by study nurses.

Nurses collected blood samples from the median antecubital vein of the anterior forearm using 21 and 23G butterfly Vacutainer^®^ brand blood collection sets (Becton Dickinson and Company, Franklin Lakes, NJ).

### Oral glucose tolerance test

Participants eligible for the OGTT were asked to consume a 75 g glucose drink within 5 minutes of having the first blood sample drawn. Two hours later, a second blood sample was taken. If the participant showed any signs or symptoms of hypoglycemia or hyperglycemia, an appropriate remedial protocol was followed.

### Hemoglobin

Hemoglobin concentration was measured either from dispensed venous blood or blood drops taken from a finger prick with the HemoCueTM 201+ portable photometer (HemoCue, Inc., Lake Forest, CA).

### Toenail clippings

Toenail clippings were collected to analyze for selenium content, an antioxidant and a marker of country food consumption. Any toenail polish was removed before collection. Toenail clippings were cut from all toenails if possible; fingernails were used if toenails were too short. All nails were placed in labeled plastic vials. Clippers and tweezers were sterilized in isopropanol.

### Forearm bone ultrasound

For women 40 years of age and older, bone density in the forearm (including both radius and ulna) was measured on the Lunar PIXI bone densitometer (GE LUNAR Corporation, Madison, WI). The non-dominant arm was tested unless it had been affected by previous fractures or arthritis, in which case the dominant arm was used. Estimated bone mineral density (BMD; g/cm^2^) and Z-score (for non-postmenopausal women under 50) or T-score (for post-menopausal women) were recorded.

### Carotid intima-media thickness

For both men and women 40 years of age or older, carotid intima-media thickness was measured by ultrasound. Intimal to medial arterial wall thickness (IMT) of the carotid artery was measured with a high-resolution B-mode ultrasound portable device (Model LogiqBook, GE Medical System, Milwaukee, WI) with a linear 4–10 MHz probe (Model 10LB-Rs, GE Medical System, Milwaukee, WI).

### Holter monitoring

Heart rate and dysrhythmias were assessed for men and women 40 years of age and older. Ambulatory electrocardiography was recorded for 2 hours with a Holter monitor SEER^®^ Light, Ambulatory Recorder/Controller (GE Medical Systems Information Technologies, Milwaukee, WI). Start time and end time were recorded on the clinical sheet. Upon completion of the recordings, data were transferred onto a portable laptop where the quality of the signal was inspected. At the end of each day, the data were backed up on an external hard drive. Daily inspection of the apparatus, including change of batteries, was performed before the beginning of the clinical investigation. A cardiologist reviewed the Holter monitoring recordings for the presence of dysrhythmias.

### Medical chart review

Three nurses traveled back to all 36 communities and completed medical chart reviews for participants who had agreed to take part in the International IHS. They verified the presence of heart disease, diabetes and related chronic medical conditions and medication usage prior to participation in the IHS.

## Blood sample preparation and analyses

On board the ship, a team of technicians prepared the blood samples for shipment to McGill University/CINE. The individual sample preparations are listed below and the individual blood tests can be found in Appendix A, Table I.

In general, blood samples were kept on ice at all times. After preparation, each vial was capped tightly, placed in a cryovial storage box and immediately frozen at −80°C. Samples in the non-coastal communities were stored at −15°C until transfer to CINE. A record of all aliquots was made. Samples were shipped frozen and placed in a locked −80°C freezer upon return to CINE, McGill University.

### Plasma preparation for glucose analysis

Two samples (at fasting and after an OGTT of blood were collected in 2.0-mL Vacutainer^®^ blood collection tubes coated with 5.0 mg of sodium fluoride and 4.0 mg of potassium oxalate (grey top – Becton Dickinson and Company, Franklin Lakes, NJ) and centrifuged as soon as possible at 2,400 rpm for 20 minutes at 4°C. Plasma was transferred into cryovials labeled either G-1 for fasting glucose or G-2 for post-OGTT.

### Plasma preparation

From each participant, 2 samples of blood were collected in 10.0-mL Vacutainer^®^ blood collection tubes coated with spray-dried K_2_ EDTA, an anticoagulant (lavender top – Becton Dickinson and Company, Franklin Lakes, NJ). Samples were taken to the laboratory every hour for processing. Two 1-mL aliquots of whole blood were taken from 1 of the vacutainers and frozen at −80°C. One 2-mL aliquot of whole blood was transferred to a 15-mL tube for isolation of platelets. Vacutainers were then centrifuged at 2,400 rpm for 20 minutes at 4°C. Plasma from both vacutainers was carefully transferred to a labeled 15-mL tube. Numerous aliquots were then dispensed into cryovials.

### White blood cell preparation

Consultations in the ISR resulted in an agreement for a special genetic research component of the International Polar Year (IPY) IHS. For this component white blood cells were prepared. Once the plasma was removed, the buffy coat was carefully transferred from both tubes to a 1.5-mL cryovial.

### Red blood cell preparation

After the plasma and buffy coat were removed, the remaining red blood cells (RBCs) were gently washed 3 times with ice cold saline (for 1 L: 8.8 g NaCl, 0.3 g EDTA, 1.0 L distilled H_2_0) and centrifuged. A 200-µL aliquot of RBCs for fatty acid analysis was transferred to a 1.5-mL cryovial and gently mixed with 200 µL of a 1:1 solution of ice-cold distilled water/methanol plus 8.4 µg butylated hydroxytoluene. Additionally, a 1-mL aliquot of RBCs was transferred to each of two 1.5-mL cryovials, flushed with nitrogen and capped tightly. In the non-coastal communities nitrogen was unavailable.

### Platelet isolation

A 2-mL aliquot of whole blood was centrifuged (200× g) for 10 minutes at 4°C. The supernatant (platelet rich plasma) was transferred into a 15-mL centrifuge tube, capped and centrifuged at 3,000×g for 30 minutes at 4°C. The resulting white pellet contained the platelets. The supernatant was decanted and the pellet carefully re-suspended in 10 mL of Na/K buffer (50 mM NaH_2_PO_4_, 5 mM KCl, 120 mM NaCl, pH 7.4). The solution was centrifuged again at 3,000× g for 30 minutes. The supernatant was discarded and the pellet re-suspended in 0.5 mL of Na/K buffer. The platelet suspension was transferred to a cryovial and stored at 4°C until transport on ice to the University of Northern British Columbia for analysis.

### Serum preparation

Three samples of blood per participant were collected in 8.5-mL Vacutainer^®^ blood collection tubes with added clot activator and gel for serum separation (red & grey top – Becton Dickinson and Company, Franklin Lakes, NJ). The vacutainers were taken to the laboratory every hour for processing. Vacutainers were then centrifuged at 2,400 rpm for 20 minutes at 4°C. Serum was transferred to a 15-mL tube and the remaining material discarded. Serum was divided into numerous aliquots and placed in cryovials.

## Data management

### Data entry

All forms, questionnaires and medical chart reviews were entered into a Microsoft Access 2003 database designed for the IHS by Solutions de formation DJH (Montreal, QC). Data were entered into the database as recorded in the questionnaires and checked for data entry errors. Results for all biomarker data were imported into the database.

Food frequency information was entered with Epi Info™ (Centers for Disease Control and Prevention Atlanta, Atlanta, Georgia) and data were double verified (2007) or double entered (2008). 24-hour dietary recall information was entered with CANDAT Software (Godin London Incorporated, London, Ontario). The 2007b Canadian Nutrient File (CNF) (25) was used to determine nutrient composition of foods. An additional in-house food file was created for foods not present in the CNF. Nutrients in these foods were obtained from food labels, recipes, and other internet resources. Nutrient values from the U.S. were checked for any fortification differences with Canadian products. All 24-hour recalls were double verified. When information on foods or portion sizes was missing from the 24-hour recall, some assumptions were made based on a documented default value. For example, if the ingredients in a food such as stew were not well described, a default food with a northern recipe was entered. Default foods/beverages were determined from 24-hour recalls where this information was provided in detail or from resources obtained from communities.

### Governance of and access to data

Our regional steering committees have full governance of and access to the data; however, McGill University/CINE maintains the database. With the original objectives of the IHS met, the 3 regional committees have merged into the IHS National Committee. This committee will continue collaboration, consultation and coordination on matters arising from the IPY – IHS, including ongoing data analyses, management of data files and requests from researchers and students for access to data, approval process for publications and conference presentations, reports, funding opportunities, knowledge translation, and intervention strategies.

## Knowledge translation and communication

Our memoranda of agreement require that all results from the IHS be submitted to steering committees representing the ISR, Nunavut and Nunatsiavut communities prior to public dissemination. Our knowledge translation activities have included a variety of approaches prior to, during and after the completion of this survey. Recruitment brochures were delivered to potential households; posters of the ship route for 2007 and 2008 were displayed in communities; an on-line travel blog during the fieldwork was posted in 2008; and the website was updated regularly. Participants received their personal results letters within 5 months of data collection. Plain language summary results covering dimensions of health, contaminants and community and personal wellness were disseminated to all participating communities, health centers, Inuit partners and organizations and funding agencies. A photographic summary has been deposited with the IPY Legacy Project. Scientific conference presentations and peer review publications (26–34) provide another means of informing Inuit stakeholders, public health professionals and policy makers.

## Conclusion

The Adult IHS 2007–2008 adds important baseline information for future comparisons and the opportunity to link with the International Inuit Cohort, a follow-up evaluation for prospectively assessing factors leading to the progression of chronic diseases.

## Appendix A

**Table I:** Laboratory analyses^a^

Sample type	Test
Whole blood	Heavy metals (Cd, Hg, Pb) Hemoglobin Selenium
Plasma	Isoprostanes (random subsample *n*=233) PCBs, toxaphene Vitamin B_6_ (subsample *n*=248)
Red blood cells (RBC)	RBC fatty acids RBC folate (random subsample of women of reproductive age *n*=249) RBC magnesium (2008 samples only *n*=1,027)
Platelets	Monoamine oxidase (MAO)
Toenail	Selenium
Serum	25(OH) vitamin D Adiponectin Apolipoprotein B Brucella Cholinesterase (ChE) Cholesterol – total C-reactive protein (hsCRP) Dioxin Responsive Chemical-Activated LUciferase gene eXpression (DR-CALUX), for women ≥40 years *n*=584 Echinococcus serology Ferritin Glucose fasting Glucose OGTT (subsample of participants *n*=834) HDL cholesterol Helicobacter pylori Insulin Leptin Osteocalcin (random subsample of women ≥40 years of age *n*=380) Paraoxonase (PON-1) Parathyroid hormone (PTH) Toxocara serology Toxoplasma serology Transferrin receptor (subsample of participants *n*=1,039) Trichinella serology Triglycerides

a For most analyses the sample size ranged from 1,147 to 2,221 unless otherwise noted.
